# A Technique for High-Throughput Protein Crystallization in Ionically Cross-Linked Polysaccharide Gel Beads for X-Ray Diffraction Experiments

**DOI:** 10.1371/journal.pone.0095017

**Published:** 2014-04-16

**Authors:** Michihiro Sugahara

**Affiliations:** RIKEN SPring-8 Center, Sayo, Hyogo, Japan; Weizmann Institute of Science, Israel

## Abstract

A simple technique for high-throughput protein crystallization in ionically cross-linked polysaccharide gel beads has been developed for contactless handling of crystals in X-ray crystallography. The method is designed to reduce mechanical damage to crystals caused by physical contact between crystal and mount tool and by osmotic shock during various manipulations including cryoprotection, heavy-atom derivatization, ligand soaking, and diffraction experiments. For this study, protein crystallization in alginate and *κ*-carrageenan gel beads was performed using six test proteins, demonstrating that proteins could be successfully crystallized in gel beads. Two complete diffraction data sets from lysozyme and ID70067 protein crystals in gel beads were collected at 100 K without removing the crystals; the results showed that the crystals had low mosaicities. In addition, crystallization of glucose isomerase was carried out in alginate gel beads in the presence of synthetic zeolite molecular sieves (MS), a hetero-epitaxic nucleant; the results demonstrated that MS can reduce excess nucleation of this protein in beads. To demonstrate heavy-atom derivatization, lysozyme crystals were successfully derivatized with K_2_PtBr_6_ within alginate gel beads. These results suggest that gel beads prevent serious damage to protein crystals during such experiments.

## Introduction

The methods and technologies for crystallization of proteins for use in X-ray diffraction experiments have advanced dramatically in recent years. However, in general, it is difficult to obtain high-quality diffraction data from every protein crystal. Regardless of the type of X-ray source, production of high-quality crystals during the crystallization process and prevention of crystal damage during crystal manipulation are essential for collection of diffraction data. Protein crystallization in gels such as agarose, agar, and silica gels is a well-known technique for production of high-quality crystals [1]–[3]; in such methods, crystal quality is improved due to reductions in convective flow, crystal sedimentation, nucleation, and twinning [2], [4], [5]. Another advantage of the gel techniques is that the gel around the crystals prevents serious damage caused by osmotic shock during ligand soaking [6]–[8].

In the conventional crystal manipulation process, manual handling with the commercial crystal mounting loops such as Hampton CryoLoop (Hampton Research), MiTeGen MicroLoop (MiTeGen LLC), and LithoLoop (Molecular Dimensions Inc.) often causes mechanical damage to the protein crystals due to physical contact between crystal and loop, resulting in the deterioration of diffraction quality. A technique for directly manipulating a gel medium that contains the crystals would minimize such risks throughout the experiment, thereby yielding superior-quality diffraction data. Protein crystallization in gel-filled capillary tubes [6], [9], gel slices [10], rehydratable gel [11], and hydrogel beads [12] have been reported, and such methods have been successfully used to crystallize proteins. These techniques can potentially prevent mechanical damage caused by physical contact with the crystals. In particular, ionically cross-linked alginate-calcium gel bead technique may allow high-throughput crystallization screening and X-ray diffraction studies in which crystals grown in gel beads are exposed to X-rays without being first removed from the beads, thus permitting acquisition of diffraction data through contactless handling of crystals.

An effective gel-bead technique for diffraction experiments would greatly facilitate protein structural research. However, the aforementioned gel-bead crystallization method is a very complicated process that could not be adapted for the high-throughput crystallization screening. Furthermore, no method has yet been established for mounting a large single crystal in a gel bead for the purpose of a diffraction experiment. Protein crystallization and crystal manipulation require a simple and versatile technique that can both improve the efficiency of crystallographic experiments and reduce errors in these complicated processes. Here, a simple protein crystallization and crystal handling technique in which all aspects of crystallographic experiments from crystallization to collection of diffraction data are accomplished in a gel bead is described.

## Materials and Methods

### Crystallization in gels

**Figure 1:**
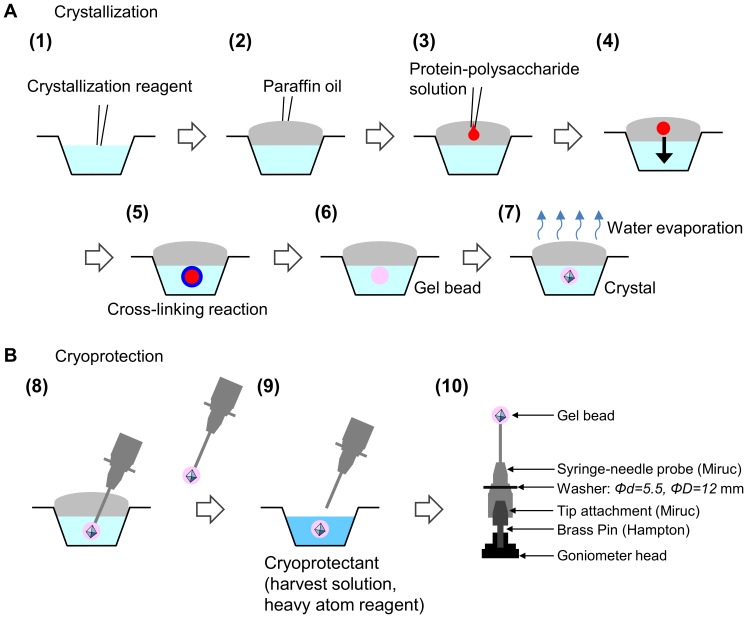
A schematic illustration of the crystallization and cryoprotection procedures in the polysaccharide gel-bead technique. (A) Crystallization processes (1–7). (B) Cryoprotection processes for data collection at 100 K (8–10).

Protein crystallization was performed in two types of ionically cross-linked polysaccharide gel beads: low-viscosity alginic acid sodium salt (Wako Pure Chemical Industries, 154725) and κ-carrageenan (Sigma, 22048). The crystallization process is illustrated in Fig. 1A. A 1.0 µl aliquot of the crystallization reagents, including calcium ions for alginate and potassium or sodium ions for κ-carrageenan, was dispensed into 72-well Nunc HLA crystal plate wells (Nalge Nunc International) [(1) in Fig. 1A]. The droplets were then covered with 20 µl of paraffin oil [(2) in Fig. 1A]. For the preparation of protein samples, equal volumes of the protein solution and polysaccharide solution [2% (*w/v*) alginate, 0.8 *M* sodium chloride or 1.5% (*w/v*) κ-carrageenan aqueous solution] were mixed at room temperature. These concentrations of the two polysaccharides were determined based on the manipulable solution viscosity for sample preparation, as well as gelation experiments. The experiments were performed for alginate and κ-carrageenan concentrations at 0.5%, 0.75%, 1.0%, 1.5%, and 2.0% (*w/v*). In the alginate stock solution, inclusion of 0.8 *M* sodium chloride prevents protein aggregation in the protein-alginate mixture. Subsequently, to prevent adherence of the protein-polysaccharide solution to the dispenser tip, 30 µl paraffin oil was added in the sample tube on top of the sample solution before dispensing. A 0.5 µl aliquot of the protein-polysaccharide solution was injected into the paraffin-oil layer above the crystallization reagents [(3) in Fig. 1A]. The protein drop migrated from the oil layer to the crystallization solution within minutes [(4) in Fig. 1A], after which the cross-linking reaction was immediately initiated [(5,6) in Fig. 1A]. After the crystallization was set up, the crystallization plate was stored at 293 K [(7) in Fig. 1A].

This study used six test proteins and precipitant solutions. For alginate gel beads: 40.0 mg ml^−1^ lysozyme from chicken egg white (Mr 14,300, isoelectric point 11.0; Sigma, L6876) and 3.0 *M* sodium chloride, 0.2 *M* calcium chloride, 0.1 *M* Bicine pH 9.0, for diffraction experiments using the crystals in the gel beads, or 5% (*w/v*) PEG 4000, 3.0 *M* sodium chloride, 0.2 *M* calcium chloride, 0.1 *M* MES pH 6.5, for a diffraction experiment using crystals on the gel-bead surface; 13.3 mg ml^−1^ catalase from bovine liver (Mr 57,600, isoelectric point 5.4; Sigma, C3155) and 10% (*w/v*) PEG 4000, 0.2 *M* calcium chloride, 0.1 *M* HEPES pH 7.5; 12.9 mg ml^−1^ a putative α-ribazole 5′-phosphate phosphatase mutant L38M from *Thermus thermophilus* HB8 (ID70102, Mr 19,600, isoelectric point 6.5) and 3.5 *M* sodium formate, 0.2 *M* calcium chloride, 0.1 *M* MES pH 5.8. For κ-carrageenan gel: 30.3 mg ml^−1^ diphthine synthase mutant Y175H from *Pyrococcus horikoshii* OT3 (ID70067, Mr 29,600, isoelectric point 5.8) and Index No. 24 (2.8 *M* sodium acetate pH 7.0; Hampton Research, HR2-144); 46.7 mg ml^−1^ conserved hypothetical protein from *Pyrococcus horikoshii* OT3 (ID11492, Mr 26,800, isoelectric point 5.3) and 2.0 *M* ammonium sulfate, 0.2 *M* potassium chloride, 0.1 *M* acetate pH 4.6. To reduce excess nucleation in the gel beads, aluminosilicate synthetic zeolite Molecular Sieves (MS, Wako Pure Chemical Industries) were examined as heteroepitaxic nucleants [13], [14]. MS with a size of 0.2–0.3 mm was prepared by passing through a series of stainless steel mesh filters of varying pore sizes. In the presence of MS, the crystallization was set up using the same procedure described above, except for the addition of MS to the protein-alginate solution. Glucose isomerase from *Streptomyces rubiginosus* (Mr 43,000, isoelectric point 3.0; Hampton Research, HR7-102) was used at 15.0 mg ml^−1^ in conjunction with a precipitant solution consisting of 10% (*w/v*) PEG 4000, 0.2 *M* calcium chloride, 0.1 *M* MES pH 6.5. The crystallization experiments in the presence of MS were repeated at least 12 times for the condition in order to confirm the reproducibility of the results. The MS-induced single crystals were observed in the eight gel beads. Lysozyme, catalase, and glucose isomerase were used without further purification. For initial screening of crystallization conditions using the gel-bead technique, the original kit (70 crystallization conditions) for alginate gel beads (Table S1) and Hampton Index kit for κ-carrageenan gel beads were mainly used.

### X-ray data collection and structure determination

Gel beads were manipulated using a combination of vacuum tweezers (PRO-SERIES PEN-VAC, Virtual Industries, inc.) and a syringe-needle probe (MIRUC OPTICAL CO., LTD., ST-C0.2). The gel bead was attached to and detached from the syringe-needle probe by applying suction and evacuation using the vacuum tweezers [(8,9) in Fig. 1B]. Next, the bead was soaked in a 6.0 µl aliquot of the cryoprotectant solution [(9) in Fig. 1B]. Subsequently, the gel bead (attached to the syringe-needle probe) was mounted on the goniometer head of a diffractometer [(10) in Fig. 1B], and then flash-cooled at 100 K under a nitrogen-gas stream. Cryoprotection of lysozyme and glucose isomerase crystals in the gel beads was carried out using 35% (*w/v*) glycerol in the corresponding precipitant solution. The gel beads were soaked in a 6.0 µl aliquot of the cryoprotectant solution for 1 minute. The ID70067 protein crystal in the gel bead and the lysozyme crystal on a gel bead were directly cryocooled under the nitrogen-gas stream without cryoprotectant treatment.

Complete diffraction data sets were collected using a Rigaku R-AXIS V image-plate detector with synchrotron radiation at BL26B1 of SPring-8, Japan [15] and an in-house Rigaku R-AXIS VII image-plate detector with Cu Kα radiation. All data were processed using *HKL-2000* [16]. All of structure determinations were performed with a fully automated approach using the program *Crank* from the *CCP4* suite [17]. Statistics for the data collected are shown in Table 1.

**Table 1:** Data-collection statistics.

Protein	Lysozyme	70067	Glucose isomerase	Lysozyme	Lysozyme	Lysozyme
Gel type	Alginate	κ-carrageenan	alginate	alginate	alginate	alginate
Comment			MS nucleant	in gel bead	on gel surface	Pt derivative
Source	SPring-8 BL26B1	SPring-8 BL26B1	SPring-8 BL26B1	in-house	in-house	SPring-8 BL26B1
Space group	*P*4_3_2_1_2	*P*4_1_2_1_2	*P*2_1_2_1_2	*P*4_3_2_1_2	*P*4_3_2_1_2	*P*4_3_2_1_2
Unit-cell parameter						
* a* (Å)	78.86	105.02	98.12	78.72	78.36	78.78
* b* (Å)	78.86	105.02	129.22	78.72	78.36	78.78
*c* (Å)	37.02	137.56	78.45	37.01	37.11	37.01
Wavelength (Å)	1.0000	1.0000	1.0000	1.54	1.54	1.0000
Resolution range (Å)	40–1.18 (1.22–1.18)	40–2.4 (2.49–2.40)	40–1.50 (1.55–1.50)	20–1.45 (1.50–1.45)	20–1.45 (1.50–1.45)	40–1.40 (1.45–1.40)
No. of unique reflections	38914 (3814)	30853 (3003)	158134 (15765)	21232 (2085)	19254 (1752)	23548 (2307)
Redundancy	22.9 (20.6)	22.8 (23.2)	5.5 (5.5)	12.9 (12.5)	12.4 (10.3)	28.2 (27.7)
Completeness (%)	99.9 (99.8)	100 (100)	99.3 (100)	100 (100)	91.4 (85.0)	99.9 (100)
*R*_merge_ (%)†	6.5 (45.2)	6.0 (58.4)	8.4 (51.9)	6.3 (49.2)	4.0 (22.0)	6.3 (40.2)
<*I*/*σ*(*I*)>	10.1 (8.2)	12.4 (7.0)	8.2 (3.6)	10.5 (4.1)	12.5 (6.3)	10.9 (8.6)
Mosaicity (°)	0.19	0.19	0.52	0.37	0.37	0.25
SAD Phasing						
Figure of merit (before solvent flattering)				0.305	0.383	0.409
Residues built with side chain (%)				96.1	98.4	96.1

Values in parentheses are for the outermost shell.

† *R*_merge_ = ∑*_hkl_* ∑*_i_* |*I_i_*(*hkl*)–<*I*(*hkl*)>|/∑*_hkl_* ∑*_i_ I_i_*(*hkl*), where *I_i_*(*hkl*) is the *i*th observation of reflection *hkl* and <*I*(*hkl*)> is the weighted average intensity for all observations *i* of reflection *hkl*.

### Derivatization

Lysozyme crystals grown in alginate gel beads under the crystallization condition described above (3.0 *M* sodium chloride, 0.2 *M* calcium chloride, 0.1 *M* Bicine pH 9.0) were subjected to heavy-atom derivatization within the gel beads. First, a gel bead was equilibrated in a 6.0 µl aliquot of harvest solution (3.0 *M* sodium chloride, 0.2 *M* calcium chloride, 0.1 *M* MES pH 6.5) for 1 hour. After equilibration, the gel bead was soaked for 24 hours in a 6.0 µl aliquot of 20 m*M* K_2_PtBr_6_ in harvest solution. Subsequently, the bead was placed in a 6.0 µl aliquot of 35% (*w/v*) glycerol in harvest solution and soaked for 1 minute. A complete diffraction data set was collected using a synchrotron source at 100 K.

## Results and Discussion

### Crystallization in gel beads

**Figure 2:**
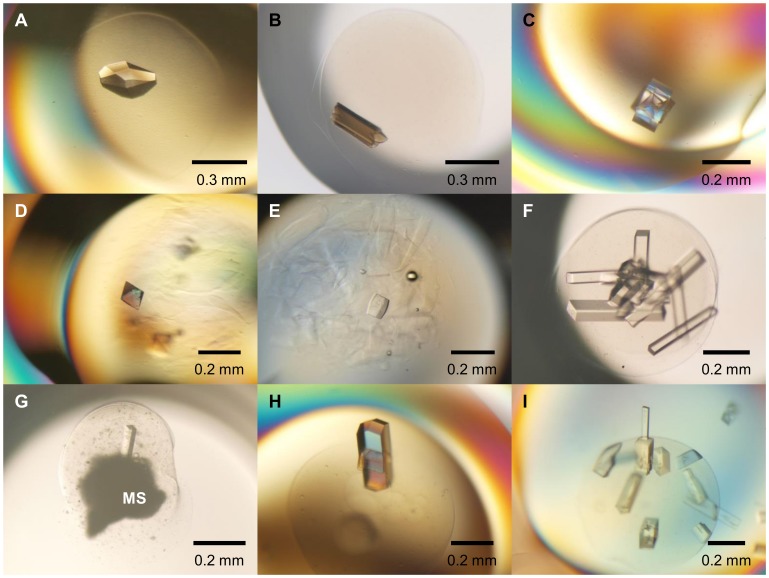
Photographs of protein crystals. (A) lysozyme, (B) catalase, and (C) ID70102 protein for alginate gel beads. (D) ID70067 and (E) ID11492 proteins for κ-carrageenan gel beads. Glucose isomerase crystals without MS (F) and with MS (G) in alginate gel beads. Crystals appeared in the gel bead after (A) 1 day, (B) 1–2 days, (C) 2–30 days, (D) 1–7 days, (E) 1 day, (F) 1 day, and (G) 1–4 days. Two proteins, (H) lysozyme and (I) glucose isomerase, crystallized in alginate gel beads after 1 day. Subsequently, additional crystals of both proteins appeared on the gel surfaces after 2–7 days.

Protein crystallization, using a combination of a technique for crystallization in hydrogel beads [12] and the oil-microbatch method [18], in ionically cross-linked alginate and κ-carrageenan gel beads was performed. The combination of these two techniques enabled us to achieve simple crystallization set-up as compared with the aforementioned hydrogel bead crystallization method (Fig. 1A). In order to evaluate the performance of this technique, the crystallization of test proteins from various sources was examined (Fig. 2). Spherically or hemispherically-shaped Gel beads with diameters of 0.5–0.9 mm were formed by dispensing 0.5 µl of the protein-polysaccharide solution. For the alginate gel beads, lysozyme, catalase, and ID70102 protein crystals of suitable size for diffraction experiments were successfully produced in the gel beads (Figs. 2A–C). The crystallization was subjected to the oil-microbatch method, which generates a concentration gradient of the reagents via slow evaporation of water in the drop. When a protein-polysaccharide solution with high viscosity is pipetted into the paraffin oil layer [(3) in Fig. 1A], it is possible to dispense a 0.5 µl droplet. The moderate viscosity of the paraffin oil used as cover oil facilitates detachment of the sample droplet from the tip during dispensing.

The alginate gel bead technique, which uses 0.2 *M* calcium chloride in the crystallization reagents, is not suitable for the structure determination of proteins that do not preferentially bind calcium ions [19], [20]. To avoid the presence of calcium ions, crystallization of ID70067 and ID11492 proteins in κ-carrageenan gel beads was performed. Crystals of both proteins in the gel beads were successfully obtained (Figs. 2D,E). κ-carrageenan beads have been used previously for immobilization and stabilization of proteins [21], [22]. In the presence of potassium ions, cross-linked κ-carrageenan forms a robust gel. Although κ-carrageenan also forms gel beads in the presence of high concentrations of sodium ions, the sodium-treated beads are much more brittle than the potassium-treated beads. Furthermore, a sodium ion concentration higher than 2.5 *M* is necessary in order to form manipulable gel beads suitable for diffraction experiments. However, in this technique, even though the gel bead in the crystallization drop is still brittle at the initial stage, it eventually becomes robust after water evaporation from the drop [(7) in Fig. 1A]. When crystallization of lysozyme in the absence of the alginate gel was carried out under the two crystallization conditions corresponding to Figs. 2A and 2H, a large number of cluster crystals with a size of 0.2–0.3 mm were observed under both conditions, implying that this bead technique is useful for optimization of crystals obtained from conventional crystallization methods. In addition, crystals from initial screening with the gel-bead technique would be optimized using a grid screening around the initial condition with variations in the buffer pH and the precipitant concentrations.

### Diffraction experiments

**Figure 3:**
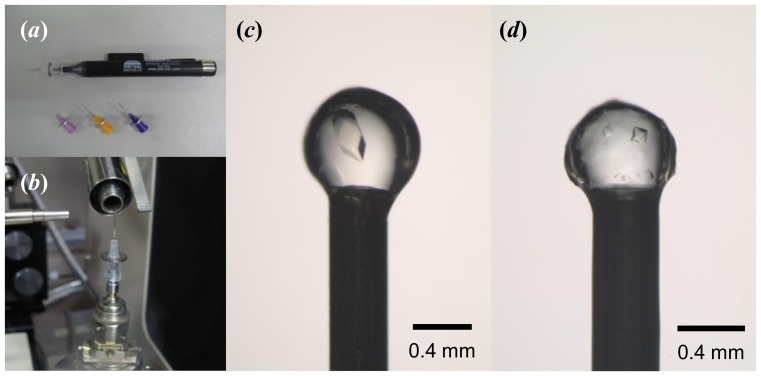
Photographs of tools for the gel-bead manipulation. (A) Vacuum tweezers and syringe-needle probes. (B) Syringe-needle probe with a gel bead mounted on the goniometer head of a diffractometer. (C) Alginate and (D) κ-carrageenan gel beads attached to the tip of syringe-needle probes.

Protein crystals grown in gel beads can be used in diffraction experiments without removing the crystals from the beads. The vacuum tweezers and the syringe-needle probe (Fig. 3A) for mounting a gel bead were chosen based on the capillary-top mounting method [23]–[25]. This innovative and simple method is suitable for capturing a gel bead in the crystallization drop well. The gel bead can be attached directly to the tip of the syringe-needle probe by applying suction using the vacuum tweezers (Figs. 3B–D). At the same time, cryoprotectant solution or paraffin oil around the gel bead can be suctioned. In order to evaluate the capabilities of this gel-bead technique, X-ray diffraction data using a synchrotron source were measured (Table 1). In all cases gel beads tightly attached to the syringe-needle probe were successfully flash-cooled at 100 K in a nitrogen-gas stream. The lysozyme crystals formed in alginate gel beads (Fig. 2A) belonged to space group *P*4_3_2_1_2, with unit-cell parameters of *a* = *b* = 78.86 Å, *c* = 37.02 Å; the crystals diffracted X-rays to 1.18 Å resolution. Sharp diffraction spots with a mosaicity of 0.19° were observed. The ID70067 protein crystals formed in κ-carrageenan gel beads (Fig. 2D) belonged to space group *P*4_1_2_1_2, with unit-cell parameters of *a* = *b* = 105.02 Å, *c* = 137.56 Å; these crystals yielded a 2.4 Å resolution data set with a mosaicity of 0.19°. The two diffraction data sets were successfully collected without making physical contact with the protein crystals. Both types of crystals had low mosaicities. The previously reported results from synchrotron experiments for tetragonal lysozyme crystals have showed that the crystals in the absence of gel media and presence of agarose gel had mosaicities 0.18–0.39° [26] and 0.17–0.24° [27], [28], respectively, indicating that the gel-bead technique is suitable for crystal production and collection of high-quality data of superior quality.

Crystallization in gels has the potential to yield crystals of superior quality because the presence of the gel medium can reduce convective flow and crystal sedimentation [2], [4], [5]. Using vacuum tweezers and the syringe-needle probe, gel beads of different sizes could be easily picked up from a variety of crystallization solutions. The crystallization method employed, in which no physical barriers are present above the crystallization droplets, is suitable for sample manipulation with easy handling. The results from the diffraction experiments indicate that this technique provides convenient capture of crystals while preventing mechanical damage during the crystal manipulation process. With conventional techniques using the crystal mounting loops such as the Hampton CryoLoop, flash cooling requires quick and trouble-free transfer of the loop-mounted crystal to the goniometer head of the diffractometer to avoid dehydration of the crystal. The present technique solves part of this problem, because the gel shields crystals from dehydration during handling. Furthermore, gel beads attached to needle-syringe probes could be stored for long periods in liquid nitrogen (Fig. S1). In X-ray crystallography, data collection at room temperature is required when protein crystals tend to crack or dissolve in cryoprotectant agents or flash-cooled crystals exhibit unacceptably high mosaicity. In this study, lysozyme crystals grown in the alginate gel beads were subjected ‘as is’ to X-ray diffraction experiments at room temperature. Unfortunately salt crystals appeared on the gel surface after approximately 5 minutes, indicating that this technique still does not apply to the data collection at room temperature. One disadvantage of κ-carrageenan gel is the beads have a rougher surface, making flash-cooled crystals poorly visible in the gel beads and hampering the alignment of the crystals in the X-ray beam (Fig. 3D). Each type of gel bead in this study has advantages and disadvantages, and application of multiple types of gel beads would be essential in order to screen a wide range of crystallization conditions.

### Control of excess nucleation

Because the beam from a synchrotron source is finely focused, it is possible to collect complete data from a single protein crystal in a gel bead without obvious scattering from the other protein crystals in the bead. The synchrotron source at micro-focus beamline BL32XU of SPring-8 [29] is useful for collecting data, especially when a gel bead includes an excessive number of protein crystals. In this bead technique, it is difficult to center the microcrystals with size less than 50 µm in the X-ray beam as compared with large crystals in the gel bead because of the optical effect from the spherical-shaped gel bead. Recently, many synchrotron beamlines have access to the crystal centering through diffraction scanning over two-dimensional grids of sample [30]–[34]. The approach may be helpful for the centering of microcrystals in gel bead. Interestingly, when the alginate-calcium gel bead with a lysozyme crystal (Fig. 2A) was soaked in a 6.0 µl aliquot of the crystallization solution (3.0 *M* sodium chloride, 0.1 *M* Bicine pH 9.0) without calcium chloride, the robust gel bead was dissolved in the solution after approximately 5 minutes. Thus, the gel media around the crystal would be easily removed from the gel bead if necessary. This may be useful for manipulation of tiny microcrystals obtained from the gel beads. On the other hand, in the case of an in-house X-ray source, it is necessary for only one large crystal to be present in the gel bead, in order to avoid additional scattering from the other crystals during data collection.

In order to reduce the excess nucleation in the gel beads, protein crystallization using synthetic zeolite Molecular Sieves (MS) was investigated. The addition of MS to an existing conventional crystallization condition promotes protein crystallization and provides higher-quality crystals in a variety of proteins [13], [14]. In some cases, MS reduced the number of nucleation sites (Fig. S2). In the absence of MS, the crystallization of glucose isomerase protein in alginate gel beads tended to yield many crystals with a size of approximately 0.05×0.05×0.3 mm as a result of excessive nucleation (Fig. 2F). Although silica gel is classified as a nucleation inhibitor [35], alginate and agarose gels act as a nucleation promoter [12], [36]. Therefore, the excess nucleation may occur in the alginate gel beads. On the other hand, in the presence of MS, a single crystal of suitable size (0.03×0.03×0.15 mm) for X-ray diffraction studies grew on the MS surface within the gel bead (Fig. 2G), indicating that the presence of MS prevents excess nucleation, but the crystal size is smaller than that for the gel bead without MS. A complete diffraction data set from the glucose isomerase crystals was collected using a synchrotron source (Table 1), demonstrating that the use of MS may allow us to skip the laborious optimization of crystallization conditions. To avoid the background signal from the powder diffraction patterns of MS, the gel bead should be mounted on the syringe-needle probe in a manner that takes into account the spindle axis of the goniometer and the relative positions of the crystal and MS. Fortunately, under a microscope, the gel bead in the crystallization drop well can be easily attached to and detached from the syringe-needle prove. The bead remount can be repeated as many times as desired, before mounting the bead on the goniometer head of the diffractometer.

### Diffraction quality

In conventional diffraction experiments, the solution inside the crystal mounting loop increases background scattering, reduces the diffraction signal-to-noise ratio, and decreases the cooling rate, degrading the quality of the data-collection statistics [37]. In this bead technique, the gel around the crystal tends to give higher background diffraction. The quality of diffraction data from the crystals in the gel beads was evaluated by sulphur single-wavelength anomalous dispersion (S-SAD) phasing using a Cu Kα X-ray source without any derivatization. A lysozyme crystal formed in an alginate gel bead yielded a 1.45 Å resolution data set with a mosaicity of 0.37° (Table 1). S-SAD and the initial model building for the protein, using the program *Crank* from the *CCP4* suite [17], automatically produced 96% complete atomic models of the protein, indicating that diffraction data adequate for structure analysis had been collected. To reduce the background noise coming from the cryocooled gel around the protein crystals and to improve the signal-to-noise ratio, small-volume dispensing is essential in order to reduce the bead size. The low thermal mass of the smaller sample size is favorable for rapid cooling [38]. In addition, the formation of amorphous ice generally requires a very fast cooling rate, because rapid cooling minimizes the increase in mosaicity of protein crystals [39]. The use of the Mosquito crystallization system (TTP Labtech), which reduces the required volume of protein sample solution to 100–200 nl, could contribute to a reduction in the size of the gel beads and high-throughput protein crystallization [3]. In this study, for the protein crystallization in the alginate gel beads, a robotic screening experiment of lysozyme was carried out using the Mosquito crystallization system. The crystallization conditions for lysozyme were screened using original 70 crystallization conditions for initial screening (Table S1). The crystallization was set up using the same procedure described above. A 1.0 µl aliquot of the crystallization reagents was automatically dispensed into 70-wells of a 96-well crystal plate. However, the dispensing of paraffin oil was performed manually because the maximum volume of the dispenser is 1.2 µl. A 0.5 µl aliquot of the lysozyme-alginate solution was successfully injected into the paraffin-oil layer by the Mosquito system. Lysozyme crystals grown in alginate gel beads were observed. This result was consistent with the experimental results from the manual crystallization, suggesting that this gel technique is adaptable to the automated crystallization of proteins.

In the alginate gel beads, additional crystals grew from the crystal surfaces of lysozyme and glucose isomerase (Figs. 2H,I). Each single crystal extended toward the outside of the gel bead, and it seems likely that the crystal growth occurred in the same direction as the seed crystals. To evaluate the diffraction quality, a lysozyme crystal on a gel bead (Fig. 2H) was directly mounted on an in-house diffractometer without undergoing a cryoprotection process. The crystal diffracted X-rays to 1.45 Å resolution with a mosaicity of 0.37° (Table 1). The background scattering level was very low as compared with that from the crystal in the gel bead (Fig. S3). The *R*_merge_ and <*I*/*σ*(*I*)> for the lysozyme crystal on the gel bead were 4.0% and 12.5, respectively, which were significantly better than those (6.3% and 10.5) for the crystal in the gel bead (Fig. 2A). When S-SAD was applied to the crystal, 98% of structure was constructed automatically with side chains. The overall figure of merit before solvent flattening was 0.383, demonstrating that the quality of diffraction obtained from a crystal on a gel bead is substantially better than the quality obtained from a crystal inside a gel bead. These results suggest that the use of a single crystal on a gel surface enabled us to acquire diffraction data with lower background noise. Crystals probably grow on gel-bead surfaces due to protein diffusion out of the gel beads immediately after the cross-linking reaction. Some proteins tend to diffuse from calcium-alginate gel beads into solutions [40]. The crystal growth on a gel surface may be facilitated at lower supersaturations, resulting in improvement of the diffraction quality [41], [42]. As compared with the crystals in the gel beads, the lysozyme crystal on the gel bead had about the same size (0.1×0.1×0.15 mm). On the other hand, the crystal size (0.03×0.01×0.17 mm) of glucose isomerase on the gel bead was smaller than that (0.07×0.07×0.17 mm) in the gel bead. Diffusion of substrates from calcium-alginate gel beads into solutions is inhibited with increasing molecular weight of the substrates [40]. Therefore, glucose isomerase with a higher molecular weight may grow the crystal with a smaller size on the gel bead surface under the lower protein concentration.

### Derivatization

In protein crystallography, ligand-soaking experiments with protein crystals are important for the preparation of heavy-atom derivative crystals for use in experimental phasing, as well as for large-scale ligand screening in pharmaceutical drug development. An experiment for heavy-atom derivatization of the protein crystals in the gel beads has not been reported yet. The applicability of the gel-bead technique to heavy-atom derivatization was examined using lysozyme crystals and a heavy-atom reagent: K_2_PtBr_6_. This diffraction experiment demonstrated successful derivatization of lysozyme crystals. The SAD phasing and initial model building, using the program *Crank* from the *CCP4* suite [17], yielded a 96% complete atomic model of the lysozyme protein (Table 1). This result suggests that the gel-bead technique is useful for rapid and effective derivatization of protein crystals, implying that the approach has great potential for general use in ligand-soaking experiments.

In most unsuccessful ligand-soaking experiments, some cracking or fragmentation of soaked crystals is observed [43]. During ligand soaking of highly sensitive crystals, formation of a multilayer poly-ion complex that completely covers the gel-bead surface may decrease the permeability of ligands into the crystals. One example of such a multilayer poly-ion complex consists of a combination of alginate as the anionic polymer and poly-lysine as the cationic polymer [44], [45].

## Conclusions

Simple protein crystallization technique in ionically cross-linked polysaccharide gel beads was demonstrated. The gel-bead technique described here may be more suitable for manipulation of very brittle crystals than other conventional techniques. Ultimately, development of an automated crystal manipulation system will require contactless handling of protein crystals. This gel-bead technique, which permits contactless handling of crystals, may therefore contribute to the development of a fully automated experimental system for protein crystallography.

## Supporting Information

Figure S1 **Photographs of the storage process of a gel bead on a syringe-needle probe in liquid nitrogen.** (A) The syringe-needle probe is mounted on a goniometer head. (B) The CryoTong is placed over the probe. (C) The probe is removed from the goniometer head. (D) The Vial Clamp is used to hold the probe in a dewar containing liquid nitrogen. (E) The probe is positioned into the CrystalCap vial. (F) The CryoCane is stored in a liquid nitrogen storage dewar.
(TIF)

Figure S2 **Photographs of protein crystals.** Streptavidin from *Streptomyces avidinii* without MS (A) and with MS (B). Glucose isomerase from *Streptomyces rubiginosus* without MS (C) and with MS (D). ID70032 protein from *Pyrococcus horikoshii* OT3 without MS (E) and with MS (F). Crystallization of the three proteins was performed using the oil-microbatch method, as described previously [13].
(TIF)

Figure S3 **X-ray diffraction patterns of lysozyme crystals.** Each crystal grown in gel bead (A) and on gel surface (B) was exposed to X-rays.
(TIF)

Table S1 **Crystallization conditions for alginate gel bead.**
(DOCX)

## References

[1] Thiessen KJ (1994) The use of two novel methods to grow protein crystals by microdialysis and vapor diffusion in an agarose gel. Acta Crystallogr D Biol Crystallogr 50: 491–495. 15299408 10.1107/S0907444994001332

[2] Robert MC, Lefaucheux F (1988) Crystal growth in gels: Principle and applications. J Cryst Growth 90: 358–367.

[3] Lorber B, Sauter C, Théobald-Dietrich A, Moreno A, Schellenberger P, et al (2009) Crystal growth of proteins, nucleic acids, and viruses in gels. Prog Biophys Mol Biol 101: 13–25. 20005247 10.1016/j.pbiomolbio.2009.12.002

[4] Garcıa-Ruiz JM, Novella ML, Moreno R, Gavira JA (2001) Agarose as crystallization media for proteins: I: Transport processes. J Cryst Growth 232: 165–172.

[5] Gavira JA, García-Ruiz JM (2002) Agarose as crystallisation media for proteins II: Trapping of gel fibres into the crystals. Acta Crystallogr D Biol Crystallogr 58: 1653–1656. 12351880 10.1107/s0907444902014609

[6] Gavira JA, Toh D, Lopéz-Jaramillo J. García-Ruiz JM, Ng JD (2002) Ab initio crystallographic structure determination of insulin from protein to electron density without crystal handling. Acta Crystallogr D Biol Crystallogr 58: 1147–1154. 12077434 10.1107/s0907444902006959

[7] Sauter C, Balg C, Moreno A, Dhouib K, Théobald-Dietrich A, et al (2009) Agarose gel facilitates enzyme crystal soaking with a ligand analog. J Appl Crystallogr 42: 279–283.

[8] Sugiyama S, Maruyama M, Sazaki G, Hirose M, Adachi H, et al (2012) Growth of protein crystals in hydrogels prevents osmotic shock. J Am Chem Soc 134: 5786–5789. 22435400 10.1021/ja301584y

[9] López-Jaramillo FJ, García-Ruiz JM, Gavira JA, Otálora F (2001) Crystallization and cryocrystallography inside X-ray capillaries. J Appl Crystallogr 34: 365–370.

[10] Garcıa-Ruiz JM, Hernández-Hernández A, López-Jaramillo J, Thomas B (2001) Crystallization screening directly in electrophoresis gels. J Cryst Growth 232: 596–602.

[11] Li Y, Guo D, Zheng B (2012) Rehydratable gel for rapid loading of nanoliter solution and its application in protein crystallization. RSC Advances 2: 4857–4863.

[12] Willaert R, Zegers I, Wyns L, Sleutel M (2005) Protein crystallization in hydrogel beads. Acta Crystallogr D Biol Crystallogr 61: 1280–1288. 16131762 10.1107/S0907444905021566

[13] Sugahara M, Asada Y, Morikawa Y, Kageyama Y, Kunishima N (2008) Nucleant-mediated protein crystallization with the application of microporous synthetic zeolite. Acta Crystallogr D Biol Crystallogr 64: 686–695. 18560157 10.1107/S0907444908009980

[14] Sugahara M, Kageyama-Morikawa Y, Kunishima N (2011) Packing space expansion of protein crystallization screening with synthetic zeolite as a heteroepitaxic nucleant. Crystal Growth & Design 11: 110–120.

[15] Ueno G, Kanda H, Hirose R, Ida K, Kumasaka T, et al (2006) RIKEN structural genomics beamlines at the SPring-8; high throughput protein crystallography with automated beamline operation. J Struct Funct Genomics 7: 15–22. 16645781 10.1007/s10969-005-9005-5

[16] Otwinowski Z, Minor W (1997) Processing of X-ray diffraction data collected in oscillation mode. Methods Enzymol 276: 307–326. 10.1016/S0076-6879(97)76066-X 27754618

[17] Collaborative Computational Project, Number 4 (1994) The CCP4 suite: programs for protein crystallography. Acta Crystallogr D Biol Crystallogr 50: 760–763. 15299374 10.1107/S0907444994003112

[18] Chayen NE, Shaw Stewart PD, Maeder DL, Blow DM (1990) An automated system for micro-batch protein crystallisation and screening. J Appl Crystallogr 23: 297–302.

[19] Han Q, Jia J, Li Y, Lollike K, Cygler M (2000) Crystallization and preliminary X-ray analysis of human grancalcin, a novel cytosolic Ca^2+^-binding protein present in leukocytes. Acta Crystallogr D Biol Crystallogr 56: 772–774. 10818361 10.1107/s0907444900005096

[20] Xie X, Dwyer MD, Swenson L, Parker MH, Botfield MC (2001) Crystal structure of calcium-free human sorcin: A member of the penta-EF-hand protein family. Protein Sci 10: 2419–2425. 11714909 10.1110/ps.36701 PMC2374028

[21] Sankalia MG, Mashru RC, Sankalia JM, Sutariya VB (2006) Stability improvement of alpha-amylase entrapped in kappa-carrageenan beads: physicochemical characterization and optimization using composite index. Int J Pharm 312: 1–14. 16500055 10.1016/j.ijpharm.2005.11.048

[22] Sankalia MG, Mashru RC, Sankalia JM, Sutariya VB (2006) Physicochemical characterization of papain entrapped in ionotropically cross-linked kappa-carrageenan gel beads for stability improvement using Doehlert shell design. J Pharm Sci 95: 1994–2013. 16850431 10.1002/jps.20665

[23] Kitago Y, Watanabe N, Tanaka I (2005) Structure determination of a novel protein by sulfur SAD using chromium radiation in combination with a new crystal-mounting method. Acta Crystallogr D Biol Crystallogr 61: 1013–1021. 16041065 10.1107/S0907444905012734

[24] Watanabe N (2006) From phasing to structure refinement in-house: Cr/Cu dual-wavelength system and a loopless free crystal-mounting method. Acta Crystallogr D Biol Crystallogr 62: 891–896. 16855305 10.1107/S0907444906010432

[25] Kitago Y, Watanabe N, Tanaka I (2010) Semi-automated protein crystal mounting device for the sulfur single-wavelength anomalous diffraction method. J Appl Crystallogr 43: 341–346.

[26] Alkire RW, Rotella FJ, Duke NEC (2013) Testing commercial protein crystallography sample mounting loops for movement in a cold-stream. J Appl Crystallogr 46: 525–536.

[27] Hasenaka H, Sugiyama S, Hirose M, Shimizu N, Kitatani T, et al (2009) Femtosecond laser processing of protein crystals grown in agarose gel. Journal of Crystal Growth 312: 73–78.

[28] Sugiyama S, Hirose M, Shimizu N, Niiyama M, Maruyama M, et al (2011) Effect of evaporation on protein crystals grown in semi-solid agarose hydrogel. Jpn J Appl Phys 50: 025502.

[29] Hirata K, Ueno G, Nisawa A, Kawano Y, Hikima T, et al (2010) New micro-beam beamline at SPring-8, targeting at protein micro-crystallography. AIP Conf Proc 1234: 901–904.

[30] Song J, Mathew D, Jacob SA, Corbett L, Moorhead P, et al (2007) Diffraction-based automated crystal centering. J Synchrotron Rad 14: 191–195. 10.1107/S0909049507004803 17317920

[31] Cherezov V, Hanson MA, Griffith MT, Hilgart MC, Sanishvili R, et al (2009) Rastering strategy for screening and centring of microcrystal samples of human membrane proteins with a sub-10 µm size X-ray synchrotron beam. J R Soc Interface 6: S587–S597. 19535414 10.1098/rsif.2009.0142.focus PMC2843980

[32] Aishima J, Owen RL, Axford D, Shepherd E, Winter G, et al (2010) High-speed crystal detection and characterization using a fast-readout detector. Acta Crystallogr D Biol Crystallogr 66: 1032–1035. 20823554 10.1107/S0907444910028192 PMC6691516

[33] Bowler MW, Guijarro M, Petitdemange S, Baker I, Svensson O, et al (2010) Diffraction cartography: applying microbeams to macromolecular crystallography sample evaluation and data collection. Acta Crystallogr D Biol Crystallogr 66: 855–864. 20693684 10.1107/S0907444910019591

[34] Hilgart MC, Sanishvili R, Ogata CM, Becker M, Venugopalan N, et al (2011) Automated sample-scanning methods for radiation damage mitigation and diffraction-based centering of macromolecular crystals. J Synchrotron Rad 18: 717–722. 10.1107/S0909049511029918 PMC3161817 21862850

[35] Vidal O, Robert MC, Boué F (1998) Gel growth of lysozyme crystals studied by small angle neutron scattering: case of silica gel, a nucleation inhibitor. J Cryst Growth 192: 271–281.

[36] Vidal O, Robert MC, Boué F (1998) Gel growth of lysozyme crystals studied by small angle neutron scattering: case of agarose gel, a nucleation promotor. J Cryst Growth 192: 257–270.

[37] Thorne RE, Stum Z, Kmetko J, O'Neill K, Gillilan R (2003) Microfabricated mounts for high-throughput macromolecular cryocrystallography. J Appl Crystallogr 36: 1455–1460.

[38] Chinte U, Shah B, DeWitt K, Kirschbaum K, Pinkerton AA, et al (2005) Sample size: an important parameter in flash-cooling macromolecular crystallization solutions. J Appl Crystallogr 38: 412–419.

[39] Kriminski S, Kazmierczak M, Thorne RE (2003) Heat transfer from protein crystals: implications for flash-cooling and X-ray beam heating. Acta Crystallogr D Biol Crystallogr 59: 697–708. 12657789 10.1107/s0907444903002713

[40] Tanaka H, Matsumura M, Veliky IA (1984) Diffusion characteristics of substrates in Ca-alginate gel beads. Biotech Bioeng 26: 53–58. 10.1002/bit.260260111 18551586

[41] Yoshizaki I, Nakamura H, Sato T, Igarashi N, Komatsu H, et al (2002) Systematic analysis of the effect of supersaturation on protein crystal quality. J Cryst Growth 237–239: 295–299.

[42] García-Ruiz JM, Otálora F (2004) Macromolecular Crystals - Growth and Characterization. In: Müller G, Métois JJ, Rudolph P, editors. Crystal Growth – from Fundamentals to Technology. Amsterdam: Elsevier. pp. 369–390.

[43] López-Jaramillo FJ, Moraleda AB, González-Ramírez LA, Carazo A, García-Ruiz JM (2002) Soaking: the effect of osmotic shock on tetragonal lysozyme crystals. Acta Crystallogr D Biol Crystallogr 58: 209–214. 11807244 10.1107/s090744490101914x

[44] Elbert DL, Herbert CB, Hubbell JA (1999) Thin polymer layers formed by polyelectrolyte multilayer techniques on biological surfaces. Langmuir 15: 5355–5362.

[45] Ai H, Jones SA, Lvov YM (2003) Biomedical applications of electrostatic layer-by-layer nano-assembly of polymers, enzymes, and nanoparticles. Cell Biochem Biophys 39: 23–43. 12835527 10.1385/CBB:39:1:23

