# Supplementary figures and images for: A Technique for High-Throughput Protein Crystallization in Ionically Cross-Linked Polysaccharide Gel Beads for X-Ray Diffraction Experiments
Source: PLoS One. 2014 Apr 16;9(4):e95017. doi: 10.1371/journal.pone.0095017 (PMC3989300; doi:10.1371/journal.pone.0095017)

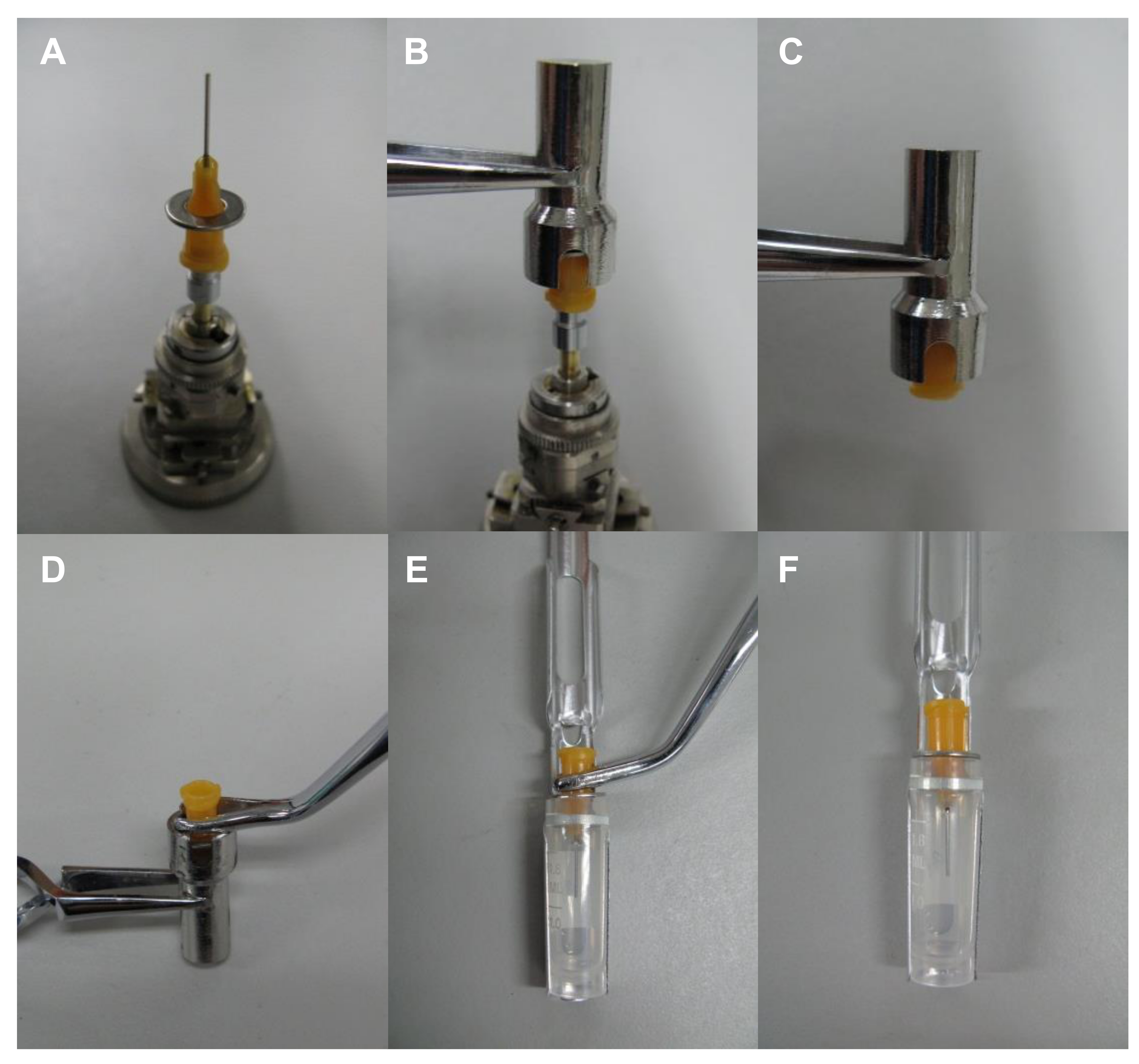

Supplement: Figure S1 — Photographs of the storage process of a gel bead on a syringe-needle probe in liquid nitrogen. (A) The syringe-needle probe is mounted on a goniometer head. (B) The CryoTong is placed over the probe. (C) The probe is removed from the goniometer head. (D) The Vial Clamp is used to hold the probe in a dewar containing liquid nitrogen. (E) The probe is positioned into the CrystalCap vial. (F) The CryoCane is stored in a liquid nitrogen storage dewar. (TIF) [file pone.0095017.s001.tif]

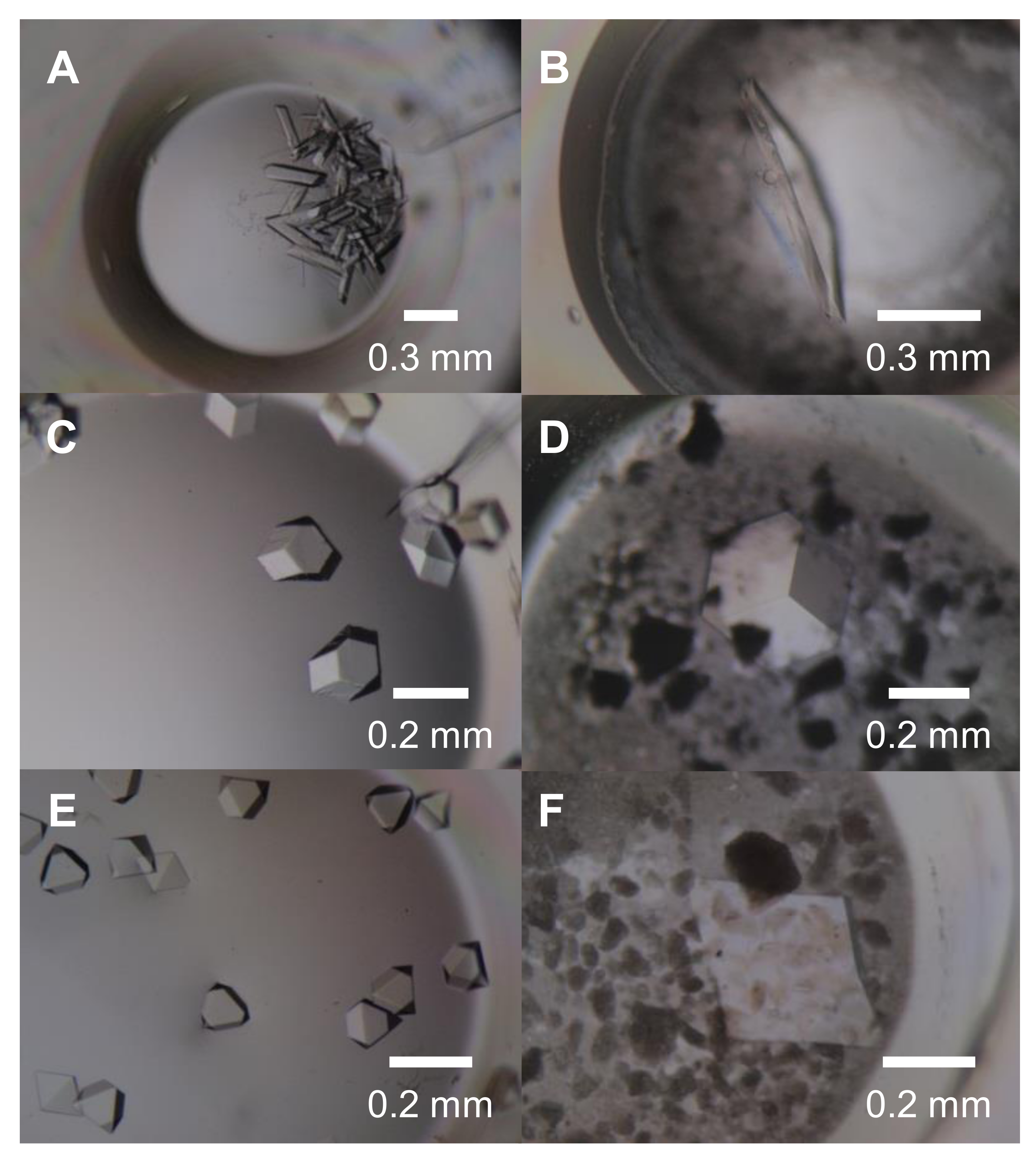

Supplement: Figure S2 — Photographs of protein crystals. Streptavidin from Streptomyces avidinii without MS (A) and with MS (B). Glucose isomerase from Streptomyces rubiginosus without MS (C) and with MS (D). ID70032 protein from Pyrococcus horikoshii OT3 without MS (E) and with MS (F). Crystallization of the three proteins was performed using the oil-microbatch method, as described previously [13]. (TIF) [file pone.0095017.s002.tif]

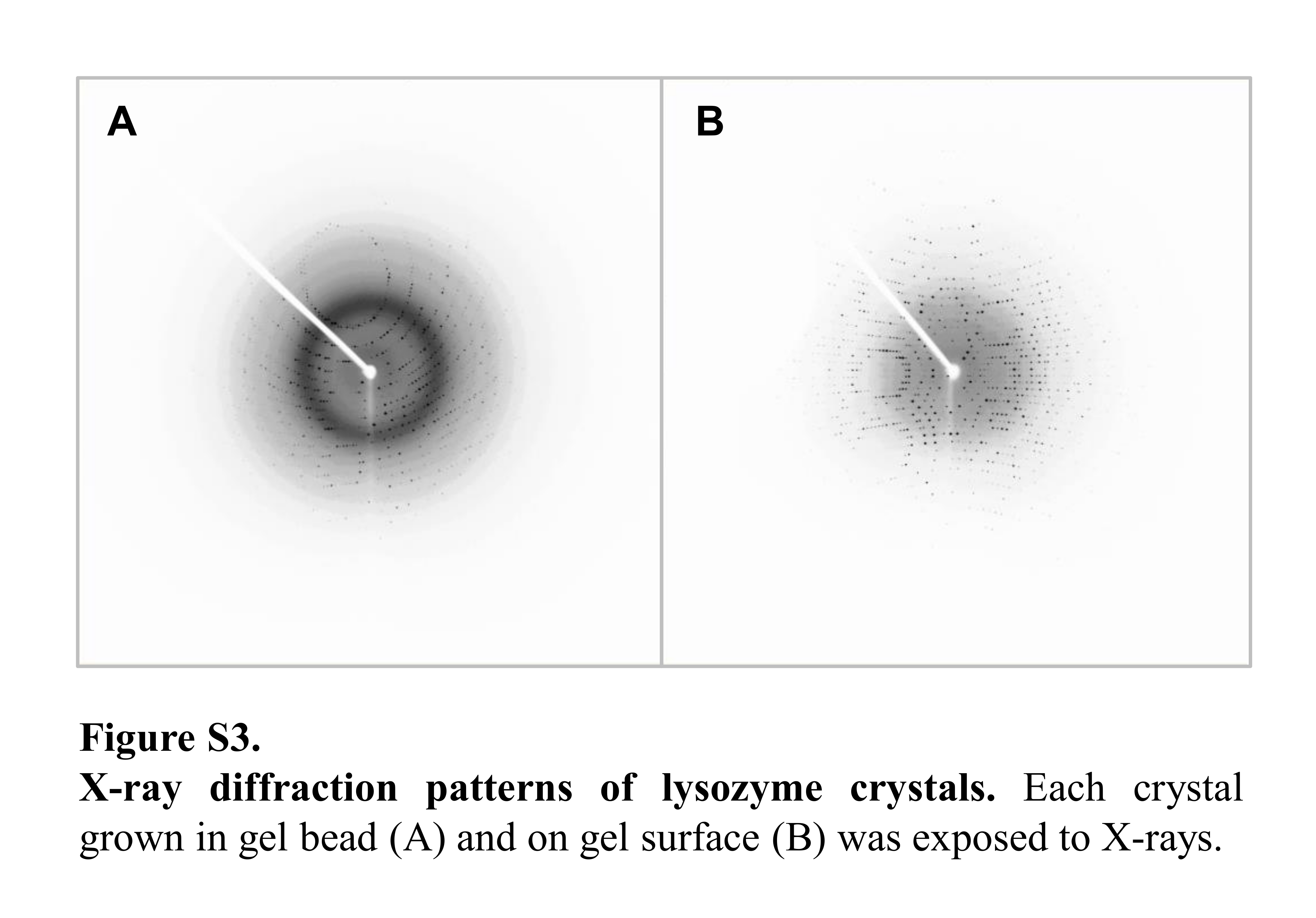

Supplement: Figure S3 — X-ray diffraction patterns of lysozyme crystals. Each crystal grown in gel bead (A) and on gel surface (B) was exposed to X-rays. (TIF) [file pone.0095017.s003.tif]
