# Supplementary material for: A Technique for High-Throughput Protein Crystallization in Ionically Cross-Linked Polysaccharide Gel Beads for X-Ray Diffraction Experiments
Source: PLoS One. 2014 Apr 16;9(4):e95017. doi: 10.1371/journal.pone.0095017 (PMC3989300; doi:10.1371/journal.pone.0095017)
Supplement: Table S1 — Crystallization conditions for alginate gel bead. (DOCX) [file pone.0095017.s004.docx]

**Table S1.** Crystallization conditions for alginate gel bead.

| No. | precipitant |  | buffer | pH | No. | precipitant |  | buffer | pH |
| --- | --- | --- | --- | --- | --- | --- | --- | --- | --- |
| 1 | 10%(v/v) iso-propanol | 0.2 M CaCl_2_ | 0.1M Acetate | 4.6 | 36 | 5%(v/v) Jeffamine M-600, 4M NaCl | 0.2 M CaCl_2_ | 0.1M Acetate | 4.6 |
| 2 | 10%(v/v) iso-propanol | 0.2 M CaCl_2_ | 0.1M MES | 5.8 | 37 | 5%(v/v) Jeffamine M-600, 4M NaCl | 0.2 M CaCl_2_ | 0.1M MES | 5.8 |
| 3 | 10%(v/v) iso-propanol | 0.2 M CaCl_2_ | 0.1M MES | 6.5 | 38 | 5%(v/v) Jeffamine M-600, 4M NaCl | 0.2 M CaCl_2_ | 0.1M MES | 6.5 |
| 4 | 10%(v/v) iso-propanol | 0.2 M CaCl_2_ | 0.1M HEPES | 7.5 | 39 | 5%(v/v) Jeffamine M-600, 4M NaCl | 0.2 M CaCl_2_ | 0.1M HEPES | 7.5 |
| 5 | 10%(v/v) iso-propanol | 0.2 M CaCl_2_ | 0.1M Bicine | 9.0 | 40 | 5%(v/v) Jeffamine M-600, 4M NaCl | 0.2 M CaCl_2_ | 0.1M Bicine | 9.0 |
| 6 | 40%(v/v) MPD^§^ | 0.2 M CaCl_2_ | 0.1M Acetate | 4.6 | 41 | 10%(v/v) PEG400, 3M NaCl | 0.2 M CaCl_2_ | 0.1M Acetate | 4.6 |
| 7 | 40%(v/v) MPD | 0.2 M CaCl_2_ | 0.1M MES | 5.8 | 42 | 10%(v/v) PEG400, 3M NaCl | 0.2 M CaCl_2_ | 0.1M MES | 5.8 |
| 8 | 40%(v/v) MPD | 0.2 M CaCl_2_ | 0.1M MES | 6.5 | 43 | 10%(v/v) PEG400, 3M NaCl | 0.2 M CaCl_2_ | 0.1M MES | 6.5 |
| 9 | 40%(v/v) MPD | 0.2 M CaCl_2_ | 0.1M HEPES | 7.5 | 44 | 10%(v/v) PEG400, 3M NaCl | 0.2 M CaCl_2_ | 0.1M HEPES | 7.5 |
| 10 | 40%(v/v) MPD | 0.2 M CaCl_2_ | 0.1M Bicine | 9.0 | 45 | 10%(v/v) PEG400, 3M NaCl | 0.2 M CaCl_2_ | 0.1M Bicine | 9.0 |
| 11 | 25%(v/v) Jeffamine M-600 | 0.2 M CaCl_2_ | 0.1M Acetate | 4.6 | 46 | 5%(v/v) PEG4000, 3M NaCl | 0.2 M CaCl_2_ | 0.1M Acetate | 4.6 |
| 12 | 25%(v/v) Jeffamine M-600 | 0.2 M CaCl_2_ | 0.1M MES | 5.8 | 47 | 5%(v/v) PEG4000, 3M NaCl | 0.2 M CaCl_2_ | 0.1M MES | 5.8 |
| 13 | 25%(v/v) Jeffamine M-600 | 0.2 M CaCl_2_ | 0.1M MES | 6.5 | 48 | 5%(v/v) PEG4000, 3M NaCl | 0.2 M CaCl_2_ | 0.1M MES | 6.5 |
| 14 | 25%(v/v) Jeffamine M-600 | 0.2 M CaCl_2_ | 0.1M HEPES | 7.5 | 49 | 5%(v/v) PEG4000, 3M NaCl | 0.2 M CaCl_2_ | 0.1M HEPES | 7.5 |
| 15 | 25%(v/v) Jeffamine M-600 | 0.2 M CaCl_2_ | 0.1M Bicine | 9.0 | 50 | 5%(v/v) PEG4000, 3M NaCl | 0.2 M CaCl_2_ | 0.1M Bicine | 9.0 |
| 16 | 15%(v/v) PEG400 | 0.2 M CaCl_2_ | 0.1M Acetate | 4.6 | 51 | 5%(v/v) PEG8000, 3M NaCl | 0.2 M CaCl_2_ | 0.1M Acetate | 4.6 |
| 17 | 15%(v/v) PEG400 | 0.2 M CaCl_2_ | 0.1M MES | 5.8 | 52 | 5%(v/v) PEG8000, 3M NaCl | 0.2 M CaCl_2_ | 0.1M MES | 5.8 |
| 18 | 15%(v/v) PEG400 | 0.2 M CaCl_2_ | 0.1M MES | 6.5 | 53 | 5%(v/v) PEG8000, 3M NaCl | 0.2 M CaCl_2_ | 0.1M MES | 6.5 |
| 19 | 15%(v/v) PEG400 | 0.2 M CaCl_2_ | 0.1M HEPES | 7.5 | 54 | 5%(v/v) PEG8000, 3M NaCl | 0.2 M CaCl_2_ | 0.1M HEPES | 7.5 |
| 20 | 15%(v/v) PEG400 | 0.2 M CaCl_2_ | 0.1M Bicine | 9.0 | 55 | 5%(v/v) PEG8000, 3M NaCl | 0.2 M CaCl_2_ | 0.1M Bicine | 9.0 |
| 21 | 10%(v/v) PEG4000 | 0.2 M CaCl_2_ | 0.1M Acetate | 4.6 | 56 | 3.0M NaCl | 0.2 M CaCl_2_ | 0.1M Acetate | 4.6 |
| 22 | 10%(v/v) PEG4000 | 0.2 M CaCl_2_ | 0.1M MES | 5.8 | 57 | 3.0M NaCl | 0.2 M CaCl_2_ | 0.1M MES | 5.8 |
| 23 | 10%(v/v) PEG4000 | 0.2 M CaCl_2_ | 0.1M MES | 6.5 | 58 | 3.0M NaCl | 0.2 M CaCl_2_ | 0.1M MES | 6.5 |
| 24 | 10%(v/v) PEG4000 | 0.2 M CaCl_2_ | 0.1M HEPES | 7.5 | 59 | 3.0M NaCl | 0.2 M CaCl_2_ | 0.1M HEPES | 7.5 |
| 25 | 10%(v/v) PEG4000 | 0.2 M CaCl_2_ | 0.1M Bicine | 9.0 | 60 | 3.0M NaCl | 0.2 M CaCl_2_ | 0.1M Bicine | 9.0 |
| 26 | 10%(v/v) PEG8000 | 0.2 M CaCl_2_ | 0.1M Acetate | 4.6 | 61 | 3.5M Sodium formate | 0.2 M CaCl_2_ | 0.1M Acetate | 4.6 |
| 27 | 10%(v/v) PEG8000 | 0.2 M CaCl_2_ | 0.1M MES | 5.8 | 62 | 3.5M Sodium formate | 0.2 M CaCl_2_ | 0.1M MES | 5.8 |
| 28 | 10%(v/v) PEG8000 | 0.2 M CaCl_2_ | 0.1M MES | 6.5 | 63 | 3.5M Sodium formate | 0.2 M CaCl_2_ | 0.1M MES | 6.5 |
| 29 | 10%(v/v) PEG8000 | 0.2 M CaCl_2_ | 0.1M HEPES | 7.5 | 64 | 3.5M Sodium formate | 0.2 M CaCl_2_ | 0.1M HEPES | 7.5 |
| 30 | 10%(v/v) PEG8000 | 0.2 M CaCl_2_ | 0.1M Bicine | 9.0 | 65 | 3.5M Sodium formate | 0.2 M CaCl_2_ | 0.1M Bicine | 9.0 |
| 31 | 10%(v/v) MPD, 1.5M NaCl | 0.2 M CaCl_2_ | 0.1M Acetate | 4.6 | 66 | 1.7M Ammonium chloride | 0.2 M CaCl_2_ | 0.1M Acetate | 4.6 |
| 32 | 10%(v/v) MPD, 1.5M NaCl | 0.2 M CaCl_2_ | 0.1M MES | 5.8 | 67 | 1.7M Ammonium chloride | 0.2 M CaCl_2_ | 0.1M MES | 5.8 |
| 33 | 10%(v/v) MPD, 1.5M NaCl | 0.2 M CaCl_2_ | 0.1M MES | 6.5 | 68 | 1.7M Ammonium chloride | 0.2 M CaCl_2_ | 0.1M MES | 6.5 |
| 34 | 10%(v/v) MPD, 1.5M NaCl | 0.2 M CaCl_2_ | 0.1M HEPES | 7.5 | 69 | 1.7M Ammonium chloride | 0.2 M CaCl_2_ | 0.1M HEPES | 7.5 |
| 35 | 10%(v/v) MPD, 1.5M NaCl | 0.2 M CaCl_2_ | 0.1M Bicine | 9.0 | 70 | 1.7M Ammonium chloride | 0.2 M CaCl_2_ | 0.1M Bicine | 9.0 |

^§^MPD: 2-methyl-2,4-pentanediol
